# Estimation of Nursing Staff in Selected Hospitals of Ilam and Ahvaz Provinces, Western Iran

**DOI:** 10.5812/nms.10605

**Published:** 2013-06-27

**Authors:** Mohammadkarim Bahadori, Mohammad Arab, Jamil Sadeghifar, Batoul Ahmadi, Mohammad Salimi, Maryam Yghoubi

**Affiliations:** 1 Health Management Research Center, Baqiyatallah University of Medical Sciences, Tehran, IR Iran; 2 Department of Health Care Management, Tehran University of Medical Sciences, Tehran, IR Iran; 3 Students’ Scientific Research Center, School of Management and Medical Information, Tehran University of Medical Sciences, Tehran, IR Iran

**Keywords:** Estimation, Nursing Staff, Hospital

## Abstract

**Background:**

The nurses as the largest group among different groups of hospital workforce play a crucial role in success of the hospital activities and promotion of community health.

**Objectives:**

This study aimed to obtain an estimation of the necessary nursing workforce status in educational hospitals affiliated to Ilam and Ahvaz University of Medical Sciences based on the scientific formula.

**Materials and Methods:**

This research was a cross sectional-descriptive study, which was done in hospitals affiliated to Ilam and Ahvaz University of Medical Sciences during 2010. Using three researcher-made forms, data was collected from all clinical, para-clinical, financial, administrative and support departments of hospitals. Data was analyzed in accordance to the standards of Iran's Ministry of Health and Medical Education using the Excel software.

**Results:**

The results showed that the nursing staffs in the 42 wards (89.5 %) were lower than the standards, only one ward (2 %) matched the standards and the rest of the wards (8.5%) were higher than the standards. The organizational dislocation (utilization of nurses in non-related jobs) of nursing staffs obtained for Imam Khomeini of Ilam, Mostafa Khomeini, Taleghani, Razi and Imam Khomeini of Ahvaz Hospitals were 8, 5, 3, 8 and 21, respectively.

**Conclusion:**

Generally, the studied hospitals were faced with a lack of nursing manpower and distribution of manpower was not appropriate. Proper planning and management of manpower in accordance with the lack of personnel, compensates and achieves the standards required for hospital’s nursing manpower and this would lead to an increase in the efficiency of hospitals activities and can provide satisfaction for the nursing staff.

## 1. Background

Hospitals in developing countries, despite covering a limited number of people, assign about 65 to 70 percent of their health sector costs to themselves (1). Besides, based on the reports provided by the Ministry of Health and Medical Education, the bed occupancy rate (BOR) of the country’s hospitals regarding active beds does not exceed 60%. The issued statistics indicate the importance of optimized use of resources and costs in the hospital sector (2). Amongst the hospitals’ operational costs, the human resources’ costs constitute the biggest share of total hospital costs. Based on previous studies and international standards, the workforce costs are estimated somewhere between 55 to 60 percent of the hospitals’ total operational costs (3). This is while the major problem of the hospitals in the country concerns the shortage of the workforce or its inadequate distribution (4). Hence, perhaps it can be said that supplying quality workforce in adequate numbers is one of the major challenges of hospital management. The workforce is among the most important hospital resources and its shortcomings and surplus play a decisive role in declining the quality of services supplied to the patients (5-8). The nurses as the largest group among different groups of hospital workforce play a crucial role in the success of hospital activities and promotion of community health (9). Providing correct and quality care and having good productivity requires a suitable number of trained and skilled nursing manpower. Different studies have noted the shortage of nursing workforce as an obstacle for the performance and quality of hospital care (10-12). Also different researches have considered the dissatisfaction of nursing personnel as a related issue to the workforce shortage (13-15). Based on the study results undertaken by Nayyeri et al. the lack of nursing workforce can to a considerable degree result in the ignorance of especial care methods and increasing mistakes, ultimately diminishing the quality of care and heightening the dissatisfaction rate of the patient (13). Other studies have shown that the shortage of nursing personnel results in the loss of up to date knowledge, motivation, severe fatigue and stress in nurses (13, 16). Recent studies are also indicative of the reverse relationship between the number of nurses and the patient’s hospitalization period (14). In this respect, it is necessary for hospitals to seriously consider their available number of nurses since its shortcomings affect different aspects of professional duties as demonstrated in numerous studies (17-19). Global average of the nursing and midwifery personnel as per 10,000 individuals is 28 people. This statistic varies for the six regions of the World Health Organization (WHO); so that the figures 68, 55, 21, 14, 11 and 11 have been reported for each 10,000 people in Europe, United State, West Pacific, Eastern Mediterranean region, Southeast Asia and Africa respectively (20). Statistics show that in public and private hospitals of Iran, ~ 70,000 nurses have been employed (considering the operation room’s personnel) (21). But most of these personnel are not officially employed due to the retirement policy issues (22). This rate in 2008 had reached 90,000 personnel in health service organizations of Iran and based on estimations, the country is in need of 220,000 nurses to provide nursing services (23). Many factors effect estimates of the nursing staff including: hours of care per patient, nursing hours available, the patients workload, leaves, absences and vacations and the various peaks of patients in each unit (24, 25). To calculate and provision nursing personnel, using a systemic attitude is required. Given the nursing labor, supplying enough number of staff would be impossible without considering the effects of other divisions, types of patients, and the required care for them as well as the non-nursing duties. The physical structure of the unit, the facilities and the equipment are among the issues affecting the number of needed personnel (26). Ghosh & Cruz in their study entitled: “Planning the nursing staff requirs a computer based model” have stated that the nursing workforce distribution in each hospital’s department depends on the type of patient, society requirements, nursing hardship for a particular patient, efficient use of nurse, care co-ordination and the organizational support (27). Hence, using an applicable and acceptable model for providing manpower, capable of determining the correct number and type of the required workforce for standard care in a health care center is an undeniable necessity (28). During the recent years, about 1.7 individuals of the official and contractual staff are considered for each active hospital bed. The cost of these staff are paid from public resources, but for the remained staffs, the hospitals are commitment to pay from exclusive incomes. This approach is not sufficient for the current requirements of hospitals and has provided abundant problems. Consequently, researches have been conducted in the country to determine the workforce index of hospitals, the most comprehensive of which being the “The State Plan of Structural Resources Allocation System of Inpatient Treatment Services” during the Fourth Development Plan of I.R.I. conducted by the Development and Promotion Bureau of Health Deputy Network of the Ministry of Health and Medical Education. Based on this project, the manpower index of a hospital is changeable depending on the bed type, bed occupancy rate (BOR), number of beds and the physical space of the hospital (29). Considering what was said above, in order to create suitable outcomes it would be necessary to determine the organizational and workforce standards for different types of hospitals (public, private, educational, non-educational) based on correct criteria, so that the hospitals could improve their performance through employing these standards and skilled managers and correct and proper use of human resources.

## 2. Objectives

Taking into consideration the geographical situation of the two provinces, Ilam and Khozestan in south eastern part of Iran and their relatively identical working conditions as well as the possibility of conducting research by the researcher, the present study has been undertaken aiming to estimate the nursing workforce status in educational hospitals affiliated to Ilam and Ahvaz University of Medical Sciences.

## 3. Materials and Methods

This descriptive cross-sectional study was conducted in the year 2010 in all educational hospitals affiliated to the Ilam University of Medical Sciences and other selected educational hospitals that are under the supervision of Ahvaz University of Medical Sciences. The statistical sample in this study included all of the wards with nursing personnel in Imam Khomeini’s hospital, Mostafa Khomeini and Taleghani of Ilam province and Razi and Imam Khomeini’s hospitals in Ahvaz. Data accumulation was performed using researcher made forms, designed using the previous studies and expert views. Three types of forms were used in this study:

Form No. 1, related to clinical staff managers through which the current nursing workforce status of these units was determined.
Form No. 2 related to the medical records department and was used for determining the bed occupancy rate (BOR), number of active beds and average length of stay of the patient in the medical wards. This form included two general areas of the hospital, including general information (eight questions) and the units’ details (equal to the number of units using workforce).
Form No. 3 was used to determine the present human resources and staff structure as well as organizational dislocation (utilization of nurses in non-related jobs) of the hospitals under study that was filled by the staff managers of the hospitals.

This form was designed for three general areas including the personnel (type of employment), organizational dislocation and the approved and occupied organizational positions. The required data was collected by one of the authors. Form Validity was approved by ten faculty members of Tehran University of Medical Sciences. Reliability in this case was not required. After accumulation of the required data, the shortages and surpluses of the nursing workforce were determined segregated by the job positions in different hospital units and the required nursing workforce was estimated. In the present study “The State Plan of Structural Resources Allocation System of Inpatient Treatment Services” has been used in which the required average time for nursing care of a patient during a 24 hours period for different units has been estimated; which means, the direct activities (setting the line, sondage, medication, etc.) or indirect activities (case registration, contacting the doctor, contacting the laboratory, etc.) a nurse would do for a patient is determined (29). The nursing workforce needed by a hospital ward was calculated by the coefficient of ward (29) (Formula 1 and 2).

Formula 1. Calculation formula for coefficient of wards based on the State Plan of Structural Resources Allocation System of Inpatient Treatment Services

Coefficient of Ward = (Direct and indirect nursing care hours × No. of days in a month) / ( Available hours for allocation to care in a month × (1- Allowance))

Formula 2. Calculation formula for the needed nursing workforce in units based on the State Plan of Structural Resources Allocation System of Inpatient Treatment Services

Needed nursing workforce = Coefficient of ward × No. of active units

- No. of days in a month = 30.5
- Available hours for allocation to care in a month (assumes 44 hours a week) = 165.12
- Allowance (permitted levels outside the optimal) = 15%
- Direct and indirect nursing care hours are shown in Table 1.

The direct and indirect nursing care hours in various clinical wards was based on a study that had been done by the Ministry of Health and Medical Education during 2010 and was obtained from the number of nurse's performance in the country. Accordingly, the average time taken to perform all required nursing care in 24 hours is based in the stopwatch method. Considering the distribution pattern of the nursing workforce according to the standards approved by the Development and Promotion Bureau of Health Deputy Network of Ministry of Health and Medical Education entitled “the State Plan of Structural Resources Allocation System of Inpatient Treatment Services”, it was determined that the shortages and surpluses of nursing workforce in each hospital related to the job position. The data were analyzed using the Excel software, descriptive analysis indexes, and manual of the used standard.

**Table 1.** Hours of Direct and Indirect Care in Wards During 24 Hours

Ward	Care duration, hours
**Internal Medicine**	4
**Psychiatry**	4
**Neurology**	4
**Heart**	4
**Gynecology**	5
**Infection Disease**	5
**Emergency Department**	8
**Oncology**	8
**Plastic Surgery**	8
**Burn**	16
**ICU**	16
**CCU**	16
**NICU**	16
**General Surgery**	4.5
**ENT**	4.5
**Eye**	4.5
**Orthopedics**	4.5
**Pediatrics**	4.5
**Urology**	4.5
**Infants**	6
**Post CCU**	6 hours a day

### 3.1. Ethical Consideration

This study was part of a research project and dissertation conducted in the School of Public Health in Tehran University of Medical Sciences. Permission to collect data from hospitals and universities was obtained. After coordination and obtaining the required permissions for gathering the data and considering different and non-integrated data in hospitals, the nursing office and hospital management’s approval of the under study hospitals were obtained for confirmation of correctness of the received data.

## 4. Results

All five hospitals under study were educational and public hospitals. The number of active beds, number of active units and average occupancy rate in each hospital has been represented in Table 2.

**Table 2.** General Data of Understudy Hospitals

Hospital	Active Beds, No.	Fixed Beds, No.	Active Units, No.	Bed Occupancy Rate, %
**Ilam**				
**Imam Khomeini**	154	200	6	67
**Mostafa Khomeini**	133	200	8	72
**Taleghani**	36	100	3	63
**Ahvaz**				
**Razi**	162	200	9	80
**Imam Khomeini**	435	570	25	78

In Imam Khomeini Hospital of Ilam, 133 organizational positions related to nursing were occupied. Based on the standards, this hospital must have had 196 organizational positions relative to the nursing staff. The most needed number of nursing staff related to the Women's Surgery ward and Men's Surgery wards with figures 15 and 25 respectively. Based on the standards and the obtained data, the number of human labor in the NICU unit was in accordance with the standards, in the infant ward it above standards and in all other units it was below standards (Table 2). Eight cases of organizational dislocation were observed for the nursing staff of this hospital. In Mostafa Khomeini Hospital, 117 organizational posts related to the nursing group were occupied. Based on the standards, this hospital must be having 166 organizational positions relative to the nursing staff. The emergency ward had surplus nursing staff based on the standards but in other wards, the hospital was faced with shortage of the nursing workforce (Table 3). Five cases of organizational dislocation were observed for nursing staff of this hospital. In Taleghani hospital, 38 approved organizational posts were occupied. This is while based on the standards; this hospital must have had 38 organizational positions relative to the nursing staff. The emergency and women’s psychiatry wards had surplus while the burn ward and men’s psychiatry wards were faced with shortage of nursing staff (Table 3). Three cases of organizational dislocations were observed for nursing staff of this hospital. In Razi Hospital, 140 organizational positions related to nursing were occupied. Based on the standards, this hospital must have had 199 organizational positions relative to the nursing staff. In all wards, the hospital was faced with a shortage of the nursing workforce. The infection disease ward requiring 11 workforces had the highest deviation from the standards (Table 3). Eight cases of organizational dislocations were observed for nursing staff of this hospital. In Imam Khomeini Hospital of Ahvaz, 376 organizational positions related to nursing were occupied. Based on the standards, this hospital must have had 627 organizational positions relative to the nursing staff. The CCU 1 ward in this hospital had surplus nursing staff based on the standards but in other wards, the hospital was faced with a shortage of nursing workforce; so that eye and orthopedics wards had the highest deviation from the standards (Table 3). Twenty-one cases of organizational dislocation were observed for the nursing staff of this hospital.

**Table 3.** The Nursing Workforce Distribution in Different Wards of Hospitals Under Study

Ward, No.	Active beds	Bed occupancy rate	Coefficient of ward	Existing status of nursing workforce	Proposed pattern	Deviation from pattern
**Razi**						
Emergency	16	-	1.73	27	28	1
Pediatrics	9	96	1.30	9	12	3
CCU	10	76	1.95	15	19	4
ICU	8	95	3.47	17	27	10
Surgery	24	68	0.97	16	23	7
Internal medicine	30	78	0.86	17	25	8
Men’s orthopedics	24	71	0.97	14	23	9
Women’s orthopedics	17	67	0.97	10	16	6
Infectious diseases	24	90	1.08	15	26	11
**Total**	162	-	-	140	199	59
**Imam Khomeini**						
Emergency	60	-	1.73	77	103	26
Pediatrics	20	96	1.3	14	26	12
CCU 1	12	87	1.95	24	23	-1
CCU 2	9	90	1.95	15	17	2
CCU 3	9	91	1.95	15	17	2
Surgery ICU	12	94	3.47	24	41	17
Internal medicine ICU	9	97	3.47	20	31	11
Heart ICU	12	61	3.47	16	41	25
NICU	9	99	3.47	17	31	14
General surgery	40	79	0.97	16	39	23
Pediatrics surgery	19	74	0.97	16	18	2
Heart surgery	16	59	0.97	13	15	2
Plastic surgery	15	-	1.56	10	23	13
Orthopedics	49	71	0.97	17	47	30
Digestive diseases	25	82	0.97	15	24	9
Eye	46	75	0.97	18	44	26
Dialysis	22	-	1.73	17	38	21
Urology	26	67	0.97	14	25	11
**Nephrology**	25	91	0.97	18	24	6
**Total**	435	-	-	376	627	251
**Imam Khomeini**						
Emergency	29	-	1.73	39	50	11
Infants	9	80	1.3	15	12	-3
ICU	5	90	3.47	15	17	2
NICU	4	85	3.47	14	14	0
Men’s surgery	45	75	0.97	18	43	25
Women’s surgery	32	85	0.97	16	31	15
Pediatrics	30	85	0.97	16	29	13
**Total**	154	-	-	133	196	63
**Mostafa Khomeini**						
Emergency	8	-	1.73	19	14	-5
CCU	8	95	1.95	13	15	2
ICU	4	95	3.47	11	14	3
Men’s Post CCU	23	95	1.3	14	30	16
Women’s Post CCU	20	90	1.3	14	26	12
Women’s surgery	23	90	0.97	16	22	6
Men’s Internal medicine	23	85	0.97	13	22	9
Women’s internal medicine	24	100	0.97	17	23	6
**Total**	133	-	-	117	166	49
**Taleghani**						
Emergency	3	-	1.73	8	5	-3
Men’s psychiatry	14	80	0.86	9	12	3
Women’s psychiatry	8	50	0.86	9	7	-2
Burn	11	60	1.3	12	14	2
**Total**	36	-	-	38	38	0

The combination and distribution of the existing as well as the needed workforce of the nursing office of the hospitals under study including nursing manager, deputy nursing manager, educational, clinical and infection control supervisors are shown in Table 4.

**Table 4.** The Staff Distribution Pattern of Nursing Managers of the Hospitals Under Study and Its Deviation from Pattern

Variables	Organizational Position. No.					
	Nursing Manager	Deputy Nursing Manager	Educational Supervisor	Clinical Supervisor	Infection Control Supervisor	Total
**Ilam**						
**Imam Khomeini Hospital**						
Existing status	1	0	1	5	1	8
Proposed pattern	1	0	1	2	1	5
Deviation from pattern	0	0	0	-3	0	-3
**Mostafa Khomeini Hospital**						
Existing status	1	0	1	3	1	6
Proposed pattern	1	0	1	2	1	5
Deviation from pattern	0	0	0	1	0	-1
**Taleghani Hospital**						
Existing status	1	0	0	1	0	2
Proposed pattern	1	0	1	2	1	5
Deviation from pattern	0	0	1	1	1	3
**Ahvaz**						
**Razi Hospital**						
Existing status	1	0	1	2	1	5
Proposed pattern	1	1	1	4	1	8
Deviation from pattern	0	1	0	2	0	3
**Imam Khomeini Hospital**						
Existing status	1	0	1	1	1	4
Proposed pattern	1	1	1	8	1	12
Deviation from pattern	0	1	0	7	0	8

## 5. Discussion

Improper management of the human resources in hospitals has irreparably damaged the hospital system of the country and many of the facilities and capacities of the hospitals that are huge investments that have been allocated to them, have remained intact (30). Based on the results, of under study hospitals, the nursing workforce combination is unbalanced and is not up to the standards. Comparison between the nursing workforce of our study and other similar studies concerning the manpower employed in hospitals such as the Sadeghifar et al study indicates the shortage of human resources in the hospitals studied in our research (31). In the Emergency care sector, competent and experienced manpower must be employed and the patient survival within the shortest amount of time must be the number one priority for measures and supply of services (32). In the present study, the emergency systems of Imam Khomeini hospitals in Ilam and hospitals of Ahvaz were in shortage of workforce and Mostafa Khomeini hospital somehow had additional personnel. It can be said that in emergency departments of Imam Khomeini hospitals in Ilam and Ahvaz hospitals despite the high number of referring patients and increase in the number of the emergency beds, the number of workforce has not been increased. However, in the two, Mostafa Khomeini and Taleghani, hospitals that were equipped with heart and burn specialized emergencies respectively, not only the lack of workforce was not an issue due to the reduced load of referrers, but also they were in excess of workforce. Rahmani et al. in a study, conducted in Tehran University of Medical Sciences hospitals, reached the conclusion that the best part of the emergency departments in the mentioned hospitals was the adequate status of management, performance, space, facilities and equipment indexes, while they experienced improper conditions concerning manpower, educational processes and instructions indexes (33). Also an international study in Babol University of Medical Sciences showed that the emergency departments of all of the understudy hospitals were faced with a shortage of workforce (34). Hence, it must be noted that the nursing workforce in this department should be based on the standard model, so that while providing effective care services in the quickest amount of time, imposing pressure on the nursing personnel could be prevented. The intensive care units (ICU) are the most indispensible and critical departments in hospitals that accommodate patients with serious situations who are vulnerable to death (35). In the present research, most of the care units (ICU, CCU and other specialized intensive care units) in the under study hospitals suffered from shortage of nursing workforce. Abrishamkar in his study concludes that the ICU departments are entangled with the lack of medical and nursing staff or failure in planning for correct use of the human resources (36). Jadidi in a study undertaken in the Markazi province hospitals reports results different from that of the previous mentioned study (35). Such contradictions are most likely not due to enough numbers of manpower, but mainly due to the hospital management policies in rotating the workforce between the departments so that the workforce shortcomings are temporarily removed to some extent. The nursing office has the role of promoting nursing services through evaluation of the present situation, determination of the quality promotion standards, consideration and recognition of the requirements and challenges of nursing, and improvement and empowerment of nursing and midwifery personnel (37). The combination of the existing nursing staff in the nursing office as part of the nursing workforce employed in the hospital is undoubtedly important. The most important point in the hospitals under study regarding the nursing office was the inexistence of educational and infection control supervisors in the Taleghani Hospital; something that can greatly harm quality of care, taking into account the important role they play and the patient type in this hospital. The organizational dislocation is a situation in which the organizational position of the employee is different from that of his current job. Considering the critical importance of the skilled and experienced manpower in hospital departments, it is of high importance that the defined job position for the employee is consistent with his/her present profession; otherwise irreparable damages will incur for the hospital and its final customers (patients) leading to a low level of satisfaction, increase in the hospital faults and turbulence of the hospital’s management. Organizational dislocation of nursing workforce in Imam Khomeini Hospital of Ilam, Mostafa Khomeini and Taleghani, Razi and Imam Khomeini of Ahvaz were determined to be 8, 5, 3, 8 and 21 respectively. It’s worth mentioning that these statistics have been gathered according to the remarks by the personnel working in various departments and labor office of the hospital that is acceptable situation according the number of nursing positions . Based on the study by Sadaqiani, due to the impossibility of providing services by having 1.7 workforce per hospital bed, the hospital managers have directly removed some of the hospital beds which sets a difference between the number of active beds and the actual capacity of the hospital beds; the outcome of this measure has decreased capacities and consequently deletion of at least 30% of services (30). In the end, it can be said that shortage in the nursing workforce and sometimes inadequate arrangement of such workforce, given that the total capacities of beds in the hospitals under study have not been utilized, will be intolerable. There is no comprehensive, consistent and integrated system for calculation of care hours in different wards of hospitals in our country. In some wards such as the emergency department, care hours depends on different factors thus standard can be somewhat different in various hospitals. Also, there is no consistent definition for needed nursing care in wards of hospital. In various studies such as this study, assumptions, input, output and used formula are different and this makes analysis difficult. Results of this study showed that the under study hospitals are facing shortages and defects in distribution of nursing workforce in different departments. In the first place, removing the problems emanating from shortage in the nursing workforce requires absorption of enough number of new trained personnel. However, in the present conditions, considering the existing difficulties in issuing employment permissions for absorption of new nursing staff, solutions must be predicted in line with removal of problems in this area using the existing number of nurses and perhaps employing a limited number of workforce. In order to resolve the problems regarding the quantity of nursing workforce, adopting a systemic attitude and considering the structure of different departments, direct and indirect nursing activities are vital (26). Given the effect of nursing workforce shortage in increasing the amount of activity of each nurse relative to the number of patients and beds, it is recommended that targeted job descriptions based on the actual needs are arranged aiming at absorbing enough number of nursing workforce and proper management of the existing human resources. Studying and evaluating the resulting changes and revising them regarding job positions, staff number and duty descriptions must be maintained at least every three years and suitable data banks of human resources segregated based on the jobs and the employees in different fields must be provided. We believe that our formula will be useful to other health care centers and hospitals regardless of the hospital size and location.
